# Radiation-Induced Angiosarcoma Following Breast Surgery: A Case Report

**DOI:** 10.70352/scrj.cr.25-0674

**Published:** 2026-03-03

**Authors:** Mutsumi Hayashi, Yukino Kobayashi, Ryoko Semba, Kanako Ogura, Fumi Murakami, Kiyomi Kimura

**Affiliations:** 1 Department of Breast Oncology, Juntendo Nerima Hospital, Tokyo, Japan; 2 Department of Human Pathology, Juntendo Nerima Hospital, Tokyo, Japan

**Keywords:** radiation-induced angiosarcoma, breast cancer, surgery

## Abstract

**INTRODUCTION:**

Radiation-induced angiosarcoma of the breast is a rare but aggressive secondary malignancy that develops several years after breast surgery and adjuvant radiation therapy. Because of its poor prognosis and diagnostic challenges, early recognition and complete surgical excision are essential for optimal management.

**CASE PRESENTATION:**

We report a 72-year-old woman who presented with multiple painful, dark purple nodules in her left breast 7 years after breast-conserving surgery and adjuvant radiation therapy for adenoid cystic carcinoma. A core needle biopsy confirmed angiosarcoma. A total mastectomy with wide skin excision, including all overlying breast skin, and split-thickness skin grafting from the left thigh was performed to ensure negative margins, which were pathologically confirmed. Surgical removal of the tumor completely relieved her breast pain. She remains recurrence-free 6 months after surgery.

**CONCLUSIONS:**

Treatment for radiation-induced angiosarcoma is primarily surgical, with mastectomy and negative margins being the standard approach. Early detection is crucial to achieve complete resection, emphasizing the importance of careful follow-up and close attention to skin changes in patients who have received breast surgery with radiation therapy.

## Abbreviation

RIAS
: radiation-induced angiosarcoma

## INTRODUCTION

Angiosarcoma of the breast is rare, accounting for less than 1% of all breast malignancies. It can arise de novo or secondarily as a consequence of prior radiation therapy following mastectomy or breast-conserving surgery.^1)^ Radiation-induced angiosarcoma (RIAS) typically manifests years after initial treatment and is characterized by rapid progression, diagnostic challenges, and limited therapeutic options due to its high malignancy.^2,3)^

Here, we present a rare case of RIAS that developed 7 years after breast-conserving surgery and radiation therapy.

## CASE PRESENTATION

**Fig. 1:**
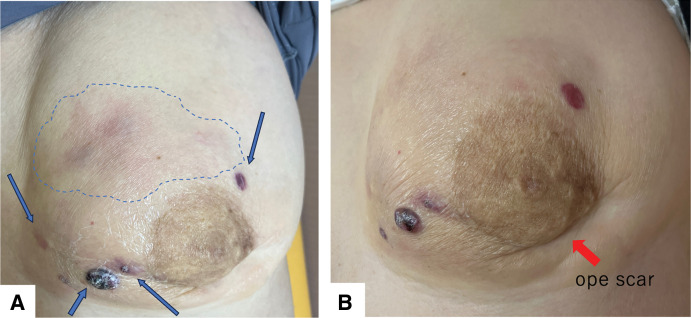
Clinical photograph at initial examination. (**A**) A dark red nodule with surrounding erythema is visible in the left breast (blue arrows and blue dotted line). (**B**) The previous surgical scar from breast-conserving surgery is also identifiable (red arrow).

**Fig. 2:**
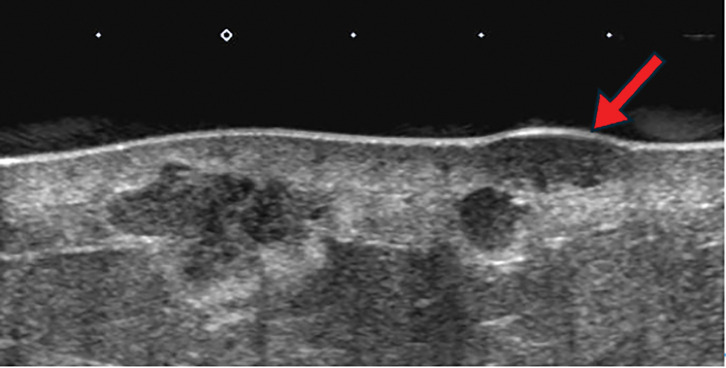
Ultrasonographic findings. Multiple irregular hypoechoic masses, some with suspected skin invasion (red arrow).

**Fig. 3:**
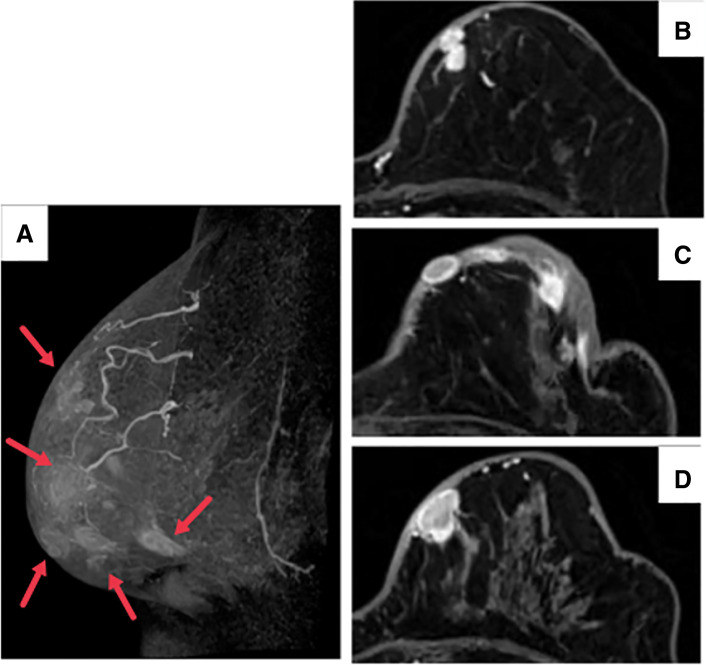
MRI findings. (**A**) Lateral 3D reconstruction showing multiple vascularized masses (red arrows). (**B**–**D**) Axial views demonstrating heterogeneous contrast enhancement.

**Fig. 4:**
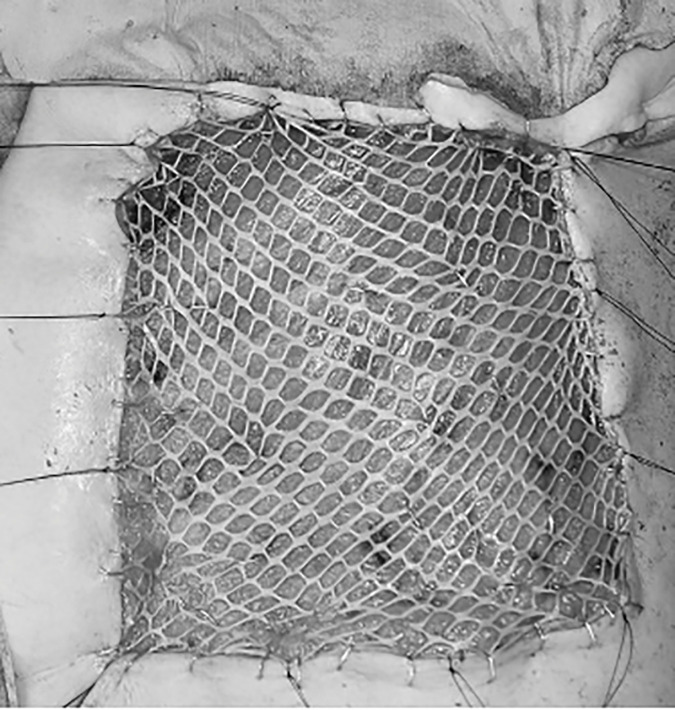
Intraoperative image. Wide skin excision of the left breast including the overlying skin, followed by split-thickness skin grafting harvested from the left thigh.

A 72-year-old woman presented with erythema, pain, and multiple dark red nodules in the left breast (**Fig. 1**). Seven years prior, she had been diagnosed with adenoid cystic carcinoma of the left breast (pT2N0M0, pStage IIA) and underwent breast-conserving surgery followed by adjuvant radiation therapy (43.2 Gy in 16 fractions). The tumor was negative for estrogen receptor, progesterone receptor, and HER2. She had no other cancer history. Her family history included esophageal cancer in her father and older brother, and lung cancer in her younger brother, with no family history of breast or ovarian cancer. For the first 5 years after surgery, she underwent follow-up with blood tests including tumor markers every 6 months, together with annual breast ultrasonography and mammography, all of which showed no abnormalities. Annual follow-up with blood tests, mammography, and ultrasonography was planned thereafter, but she failed to attend the scheduled visit in the 6th postoperative year for personal reasons. She presented for her 7th-year examination, during which she reported a 1-month history of left breast pain that had prompted her to seek evaluation. At the 7-year follow-up visit, she returned for routine examination, during which breast ultrasonography revealed multiple superficial hypoechoic masses, measuring up to 17 mm in diameter, with evidence of skin invasion (**Fig. 2**). These masses were associated with pain. A core needle biopsy of one of the nodules confirmed angiosarcoma. MRI showed multiple masses with heterogeneous enhancement (**Fig. 3**), and contrast-enhanced CT revealed no distant metastases. In addition, MRI demonstrated severe skin edema and thickening; however, the relationship between these skin changes and the angiosarcoma was unclear at that time. The disease was considered surgically resectable, and a total mastectomy with wide skin excision was subsequently performed. To ensure negative margins, all overlying skin of the left breast was excised, followed by split-thickness skin grafting harvested from the left thigh (**Fig. 4**). Preoperative imaging suggested that the angiosarcoma was confined to the subcutaneous tissue and breast parenchyma without evidence of invasion into the pectoralis major muscle. Therefore, resection of the pectoralis major was deemed unnecessary, and the muscle was preserved during surgery.Postoperatively, the patient’s severe breast pain completely resolved, likely because removal of the tumor relieved the local inflammatory and mechanical effects causing the pain. Although pain improvement after resection is not universally described in angiosarcoma, symptomatic relief can occur when the tumor is completely removed.

**Fig. 5:**
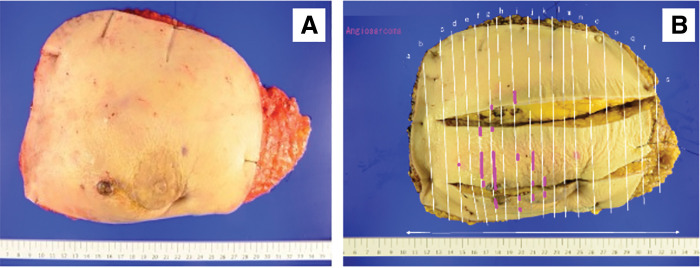
Macroscopic findings. (**A**) The entire left breast and overlying skin were excised en bloc. A nodule is exposed on the lateral side of the nipple. (**B**) Mapping image. Two incisions were made directly above the tumor before formalin fixation. The tumor extensively infiltrates the surrounding tissue.

**Fig. 6:**
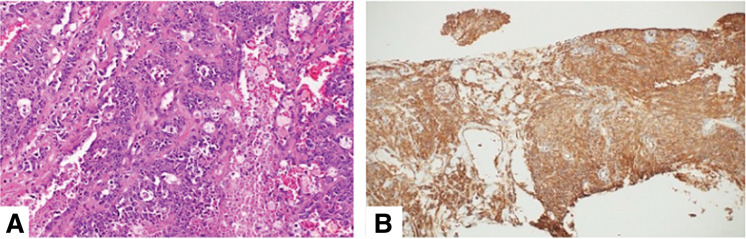
Histopathologic findings. (**A**) Hematoxylin and eosin staining showing numerous poorly differentiated spindle-shaped tumor cells with endothelial proliferation into the vascular lumen. (**B**) CD31 immunohistochemistry showing strong membranous positivity in vascular endothelial cells, supporting endothelial differentiation.

Pathologic examination showed multiple non-contiguous lesions extending over a 90-mm area, with a maximum tumor size of 27 mm, which were multifocally distributed in the subcutaneous tissue and did not correspond to the surgical scar. No sarcoma was identified in the area of skin thickening. Therefore, the severe skin edema and thickening observed on MRI were considered to be associated with postoperative and radiation-related changes following the partial mastectomy performed 7 years earlier, rather than direct tumor involvement. Surgical margins were negative (**Fig. 5**). Histologically, the tumors consisted of numerous poorly differentiated spindle-shaped cells. Prominent endothelial nuclei protruding into the vascular lumen were observed. Immunohistochemically, CD31, a specific marker for vascular endothelial cells, was positive (**Fig. 6**).

Although postoperative adjuvant chemotherapy with paclitaxel was proposed, the patient preferred close observation, and systemic treatment was not administered. She remains recurrence-free 6 months after surgery under close observation.

## DISCUSSION

RIAS typically occurs in elderly women several years after breast-conserving surgery with radiation therapy.^4,5)^ It frequently presents as multifocal lesions and has a poor prognosis due to its aggressive nature. One study reported a mean age of onset of 72 years and a latency of 7.5 years after radiation therapy, similar to the present case.^4)^ The estimated incidence of RIAS is 0.05% at 10 years and 0.5% at 30 years post-treatment.^2)^ The reported 5- and 10-year overall survival rates are 42% and 27%, respectively.^2)^

Primary angiosarcoma of the breast and RIAS share similar histopathological features but differ in several important clinical aspects. Primary angiosarcoma typically occurs in younger patients and arises within the breast parenchyma, often presenting as a rapidly enlarging, painless mass.^6)^

In contrast, RIAS develops in previously irradiated skin and subcutaneous tissue, usually more than 5 years after radiotherapy, and frequently manifests as discoloration, ecchymosis-like lesions, or multiple superficial nodules.^6)^ These distinct clinical presentations are essential for differentiating the two entities, particularly in patients with a history of breast surgery and radiotherapy.

In our case, both the patient’s age at onset (72 years) and the latency period after radiotherapy (7 years) were consistent with the averages reported in previous series.^4)^

The clinical presentation of multiple dark red nodules with associated pain also matched the typical features of RIAS. Histopathologic and immunohistochemical findings were characteristic, showing poorly differentiated spindle-shaped cells with strong CD31 positivity. However, unlike many reported cases in which the lesions arise in close proximity to the surgical scar, the tumors in our patient were multifocally distributed in the subcutaneous tissue without correspondence to the prior incision site. This distribution pattern may reflect variations in the field of radiation injury or tumor origin within the irradiated tissue.

To date, complete surgical resection is considered to be the standard procedure for RIAS.^5)^ Previous reports have described high rates of local recurrence even after complete resection. In several series, the median interval to local recurrence was approximately 1 year, and early relapse within the first year after surgery was frequently observed.^7)^ Distant metastases have also been reported, often appearing after local recurrence, with a median time of roughly 1 year from diagnosis^8)^ These clinical features indicate the aggressive behavior of RIAS and highlight the importance of early recognition, wide surgical excision, and careful postoperative surveillance.

Therefore, early diagnosis is essential to ensure sufficient resection margins and improve outcomes. Careful follow-up with close attention to skin changes is essential for patients who have received breast surgery with radiation therapy, given the potential risk of developing RIAS. In this case, the patient did not undergo the scheduled examination in the 6th postoperative year, and the possibility of having missed an opportunity for early detection cannot be ruled out.

The efficacy of adjuvant chemotherapy for angiosarcoma, including radiation-induced angiosarcoma, has not been clearly established, and its role remains a matter of ongoing debate. Nevertheless, paclitaxel remains one of the most active systemic agents for angiosarcoma. A real-world retrospective analysis of 75 patients showed an objective response rate of 23.7% and a median overall survival of 10.2 months with weekly paclitaxel as first-line therapy, particularly in cases without liver involvement.^9)^

In addition, Japanese real-world data on cutaneous angiosarcoma have supported the practical utility of taxane-based chemoradiotherapy, demonstrating acceptable safety and comparable survival outcomes.^10)^ Although these findings support the use of paclitaxel as a reasonable systemic option for high-risk cases, the benefit of adjuvant chemotherapy after complete resection remains uncertain.^11)^ In the present case, adjuvant paclitaxel was discussed based on these data; however, the patient chose close observation after surgery.

Because only 6 months have passed since surgical resection for RIAS in the present case, careful surveillance remains essential. Previous reports have shown that RIAS frequently recurs early, with most local recurrences occurring within the first 6–12 months after surgery of RIAS, and some series reporting a median time to recurrence of approximately 6–7 months.^4,7)^ Recurrence typically presents as new multifocal cutaneous or subcutaneous lesions in the irradiated field, while distant metastases to the lungs, liver, or bone may follow.^6)^ Given these recurrence patterns and the aggressive nature of RIAS, long-term close follow-up is mandatory even after complete resection.

In addition to prior radiotherapy, several factors have been discussed in the literature as possible contributors to breast radiation-associated angiosarcoma, including older age, higher radiation dose, and larger irradiated volume.^6)^ Chronic radiation-related skin and subcutaneous tissue changes, such as fibrosis or persistent discoloration, have also been suggested to predispose to malignant endothelial transformation.^12)^ However, given the rarity of RIAS and the heterogeneity of reported cases, these remain speculative rather than definitive risk factors. Therefore, when performing long-term surveillance in patients treated with breast surgery followed by radiotherapy, clinicians should consider these potential modifiers in addition to monitoring for new cutaneous lesions.

## CONCLUSIONS

We report a rare case of RIAS that developed 7 years after breast-conserving surgery and adjuvant radiation therapy. Given its aggressive course and limited treatment options, complete surgical resection at an early stage is essential. This case highlights the importance of long-term surveillance in patients undergoing breast surgery with adjuvant radiotherapy.
